# Evaluation of Nutritional Status in Geriatric Patients Before and After the Insertion of Complete Dentures

**DOI:** 10.7759/cureus.107203

**Published:** 2026-04-16

**Authors:** Arvind K Singh, Shruti S Grover, Pradeep K Pandey, Uzma Khan, Surya Mishra

**Affiliations:** 1 Department of Prosthodontics, Chandra Dental College and Hospital, Barabanki, IND

**Keywords:** diet, elderly people, food, nutrients, prosthetic rehabilitation, tooth loss

## Abstract

Objective: This study aimed to longitudinally evaluate the effect of complete denture rehabilitation on the nutritional status of completely edentulous geriatric patients by assessing dietary intake, biochemical markers, and anthropometric parameters at baseline and at one, three, and six months after insertion.

Methods: This study was designed as a prospective longitudinal (before and after) clinical study and included 30 completely edentulous geriatric male patients selected from the Department of Prosthodontics, Chandra Dental College and Hospital, Barabanki. The same cohort of participants was followed over time, with each subject serving as their own control. Nutritional status was assessed at baseline (before denture insertion) and subsequently at one, three, and six months after insertion. The assessment parameters included body mass index (BMI) and biochemical investigations, including serum calcium, vitamin B12, high-density lipoprotein (HDL), and hemoglobin (Hb) levels. Statistical analysis was performed using repeated-measures comparisons to evaluate changes over time, with the level of significance set at p < 0.05.

Results: All evaluated parameters, including Hb, serum calcium, vitamin B12, serum HDL, and BMI, demonstrated a slight, nonsignificant decrease at one month following denture insertion. However, a progressive and statistically significant increase was observed at three and six months (p < 0.001). This trend suggests an initial adaptation phase to newly inserted dentures, followed by improved masticatory efficiency and enhanced dietary intake.

Conclusion: Within the limitations of this study, complete denture rehabilitation significantly improved the nutritional status of geriatric, completely edentulous male patients over six months. Notable improvements were observed in key biochemical and anthropometric parameters, indicating the positive systemic impact of prosthodontic rehabilitation.

## Introduction

Healthy aging has emerged as a major global public health priority, with an increasing emphasis on maintaining independence, functional ability, and overall well-being among older adults [1]. Adequate nutrition plays a pivotal role in preserving systemic health, immune competence, and quality of life in the geriatric population. However, dietary intake commonly declines with advancing age due to physiological, functional, and psychosocial changes, thereby increasing the risk of nutritional deficiencies and associated morbidities [1].

Advances in medical science have contributed to increased life expectancy; nevertheless, longevity does not necessarily equate to improved quality of life. Aging is accompanied by physiological alterations that predispose individuals to chronic systemic diseases such as diabetes mellitus, hypertension, and infectious conditions, including tuberculosis, particularly in the presence of malnutrition or inadequate dietary intake [2]. In prosthodontic patients, nutritional status directly influences oral mucosal health, which in turn affects the retention, stability, and long-term success of complete denture therapy. Compromised mucosal integrity may contribute to discomfort, poor adaptation, and eventual treatment failure [2].

Tooth loss remains highly prevalent among the elderly and significantly impairs masticatory efficiency. Reduced chewing ability alters food selection and dietary patterns, often leading to avoidance of fibrous fruits, vegetables, and protein-rich foods that require greater masticatory effort [3]. Inadequate mastication may lead to swallowing of larger food particles, digestive disturbances, altered taste perception, and psychological consequences such as reduced self-esteem and depression [3]. Thus, edentulism extends beyond oral disability and may adversely affect general health and nutritional status.

The primary objective of prosthodontic rehabilitation in edentulous patients is to restore oral function, comfort, and esthetics, thereby enhancing overall health. However, complete denture wearers generally exhibit lower masticatory performance than individuals with natural dentition [4]. This reduction in masticatory efficiency can restrict dietary choices and compromise nutritional adequacy. In recent years, increasing attention has been directed toward understanding the interrelationships among nutrition, systemic health, and oral rehabilitation in elderly individuals [4].

A balanced diet and adequate physical activity are essential strategies for promoting healthy aging. Poor dietary practices in older adults contribute to sarcopenia, functional decline, immune suppression, and progression of chronic diseases [5]. In the context of complete denture therapy, dietary counseling and nutritional support may enhance mucosal tolerance, facilitate adaptation to new dentures, and improve treatment acceptance. As denture fabrication involves multiple clinical visits, nutritional assessment and guidance can be conveniently integrated into routine prosthodontic care [5].

Individuals with compromised dentition often prefer soft, carbohydrate-rich, and high-fat foods while avoiding harder, nutrient-dense foods such as meat, fruits, and vegetables [6]. Such dietary modifications may result in either undernutrition or obesity, both of which represent significant public health concerns. Reduced masticatory efficiency has been associated with altered body mass index (BMI) and suboptimal nutritional biomarkers among elderly populations [6].

Assessment of nutritional status requires a multidimensional approach. Clinical examination of integumentary structures may reveal signs of micronutrient deficiencies, while anthropometric indices such as BMI provide an estimate of body composition [7-10]. Biochemical investigations including serum hemoglobin (Hb), calcium, vitamin B12, and lipid profile parameters such as high-density lipoprotein (HDL) offer objective indicators of systemic nutritional status [6].

Age-related reductions in muscular strength prolong the duration and increase the effort of mastication. Furthermore, decreased salivary flow compromises bolus formation and swallowing efficiency. Age-associated renal changes may predispose elderly individuals to dehydration and electrolyte imbalances, further impacting overall nutritional status [11]. These physiological alterations underscore the importance of evaluating systemic health outcomes in geriatric prosthodontic patients.

Despite the recognized association between edentulism and nutrition, conventional assessment of complete denture therapy predominantly focuses on retention, stability, and esthetics. Limited evidence exists regarding its impact on objective nutritional and biochemical parameters. Therefore, evaluating changes in anthropometric and hematological markers before and after complete denture insertion may provide a broader understanding of the systemic benefits of prosthodontic rehabilitation.

The present study was undertaken to assess the nutritional status of geriatric, completely edentulous patients before and after complete denture insertion using anthropometric and biochemical parameters. By correlating functional rehabilitation with measurable systemic outcomes, this study aims to expand the scope of denture evaluation beyond conventional clinical parameters.

## Materials and methods

Methodology

The present study was conducted in the Department of Prosthodontics, Chandra Dental College and Hospital, Barabanki. Ethical approval was obtained from the Institutional Ethical Committee (CDCH/EC/PROSTH/2023-26/020) before commencement of the study, and written informed consent was secured from all participants.

Study design and participants

A total of 30 completely edentulous male patients were recruited from the outpatient department for this prospective longitudinal (before and after) clinical study using stratified random sampling. The sample size was determined before based on the expected mean difference (effect size) in vitamin B12 levels between baseline and six months after complete denture insertion, as this parameter was considered the primary outcome variable. The estimation was further supported by a previously published study with a similar study design and outcome measures that also used a sample size of 30 subjects [12].

Assuming a mean difference (µ) of 3.72 pg/mL and a pooled standard deviation (SD; σ) of 5.14 pg/mL, with a significance level of 5% (α = 0.05) and statistical power of 80% (1−β = 0.80), the minimum required sample size was calculated using the following formula:

$n = (Z_{1-\alpha/2} + Z_{1-\beta})^2 \times \frac{2\sigma^2}{\mu^2}$

$\text{where } Z_{1-\alpha/2} = 1.96 \text{ and } Z_{1-\beta} = 0.84$

$n = (1.96 + 0.84)^2 \times \frac{2(5.14)^2}{(3.72)^2}$

$n = 29.93 \approx 30$.

Thus, a minimum of 30 subjects was required to achieve the desired effect size with adequate statistical power, and accordingly, 30 participants were included in the study. Participants were aged between 55 and 70 years, had been completely edentulous for at least one year, and were receiving complete dentures for the first time. Only male participants were included to minimize potential confounding due to gender-related physiological and hormonal variations, particularly in parameters such as body composition and hematological indices, which could influence nutritional assessment outcomes. To further reduce variability, all participants were selected from a similar socioeconomic background. Patients presenting with any local or systemic debilitating disease, or those unwilling to participate or comply with the follow-up schedule, were excluded. The same cohort was followed longitudinally at predefined intervals for outcome assessment.

Study protocol

The study protocol was designed based on previously published methodologies assessing the impact of complete denture rehabilitation on the nutritional status of geriatric edentulous patients [12]. No separate nutritional screening tool was used for sample selection before enrollment; however, baseline nutritional status was comprehensively assessed for all participants at the start of the study using validated anthropometric and biochemical parameters. Nutritional status was evaluated at four time points: before complete denture insertion (baseline) and at one, three, and six months after insertion. The assessed parameters included BMI, Hb, serum calcium, serum HDL, and serum vitamin B12 levels. Baseline measurements were recorded following a comprehensive clinical examination and diagnosis.

All complete dentures were fabricated by a single experienced operator using standardized clinical protocols to minimize operator-related variability and ensure consistent treatment outcomes. After insertion, participants were recalled for follow-up evaluations at the specified intervals, during which the same anthropometric and biochemical investigations were repeated.

Anthropometric assessment

Body weight was measured using a calibrated weighing scale (Omron HN-289, Omron Healthcare India Pvt. Ltd., Gurugram, India), and height was recorded using a stadiometer (SECA 213, SECA GmbH, Hamburg, Germany). BMI was calculated as body weight in kilograms divided by the square of height in meters (kg/m²), according to the World Health Organization criteria [13].

$\mathrm{BMI} \; (\mathrm{kg/m^2}) = \frac{\text{Weight (kg)}}{\text{Height (m)}^2}$

Biochemical and hematological assessment

Venous blood samples were collected under aseptic conditions from the antecubital vein using sterile disposable syringes. Samples intended for Hb estimation were collected in ethylenediaminetetraacetic-containing tubes (BD Vacutainer®, Becton Dickinson Pvt. Ltd., Gurugram, India), while samples for serum calcium, serum HDL, and vitamin B12 estimation were collected in plain vacutainer tubes (BD Vacutainer®, Becton Dickinson Pvt. Ltd.) and centrifuged to obtain serum. Hb estimation was performed using an automated three-part differential hematology analyzer (Erba Mannheim, Transasia Bio-Medicals Ltd., Mumbai, India) based on the electrical impedance principle and cyanide-free colorimetric detection. Serum calcium analysis was carried out using a semiautomated chemistry analyzer (Erba Chem 7, Transasia Bio-Medicals Ltd., Mumbai, India) employing spectrophotometric methods. Serum HDL cholesterol was measured using an automated clinical chemistry analyzer (Erba EM 200, Transasia Bio-Medicals Ltd.) based on photometric analysis of absorbance changes. Serum vitamin B12 levels were estimated using a fully automated chemiluminescence immunoassay analyzer (MAGLUMI 800, Snibe Co. Ltd., Shenzhen, China), utilizing magnetic microbead separation and acridinium ester-based detection. All biochemical analyses were performed in accordance with manufacturer guidelines, and internal quality control procedures were maintained throughout the study period.

Outcome measures

Primary outcome measures included changes in Hb, serum calcium, serum HDL, vitamin B12, and BMI at baseline, one month, three months, and six months following complete denture insertion.

Statistical analysis

Data were summarized as mean ± SD. Normality of data distribution was assessed using the Shapiro-Wilk test, and homogeneity of variance was evaluated using Levene’s test. Repeated-measures analysis of variance (ANOVA) was used to assess changes across time intervals. Post hoc comparisons were performed using Tukey honestly significant difference (HSD) test. Pearson correlation analysis was conducted to determine relationships between variables. A two-tailed p value of <0.05 was considered statistically significant. Statistical analyses were performed using the Statistical Package for the Social Sciences software (version 22.0, IBM Corp., Armonk, NY).

## Results

The present study was conducted to evaluate and assess the nutritional status of geriatric patients before and after the insertion of complete dentures. A total of 30 completely edentulous, systemically healthy male patients were included based on predefined inclusion and exclusion criteria. Participants were aged between 55 and 70 years, had been completely edentulous for at least one year, were first-time denture wearers, and had a similar socioeconomic status (SES). Patients with any local or systemic debilitating disease, those on medications that could influence nutritional status, or those unwilling to participate or comply with follow-up were excluded.

The baseline characteristics of the study participants are presented in Table 1. A total of 30 completely edentulous geriatric patients were included in the study. Based on age distribution, 12 participants (40.0%) were ≤60 years, whereas 18 participants (60.0%) were >60 years. All study participants were men (100%). Regarding the duration of edentulism, 15 participants (50.0%) had been edentulous for ≤15 months, while the remaining 15 participants (50.0%) had been edentulous for >15 months. The mean Hb level among the participants was 13.14 ± 0.81 g/dL, while the mean serum calcium level was 8.54 ± 0.44 mg/dL. The mean vitamin B12 level was 342.93 ± 72.66 pg/mL, and the mean serum HDL level was 39.65 ± 4.22 mg/dL. Regarding BMI status, 18 participants (60.0%) were categorized as underweight (<18.5 kg/m²), whereas 12 participants (40.0%) had a normal BMI (18.5-25.0 kg/m²).

**Table 1:** Baseline characteristics of study participants (n = 30) SD: standard deviation; HDL: high-density lipoprotein; BMI: body mass index

Variable	Category	Total subjects, n (%)/mean ± SD
Age (years)	≤60 years	12 (40)
	>60 years	18 (60)
Sex	Male	30 (100)
Duration of edentulism (months)	≤15 months	15 (50)
	>15 months	15 (50)
Hemoglobin (g/dL)	-	13.14 ± 0.81
Serum calcium (mg/dL)	-	8.54 ± 0.44
Vitamin B12 (pg/mL)	-	342.93 ± 72.66
Serum HDL (mg/dL)	-	39.65 ± 4.22
BMI status	Underweight (<18.5 kg/m²)	18 (60)
	Normal weight (18.5-25.0 kg/m²)	12 (40)

The pretreatment (baseline) and postdenture Hb levels at one, three, and six months are summarized in Table 2. The mean Hb levels at baseline, one month, three months, and six months were 13.14 ± 0.81, 13.05 ± 0.82, 13.69 ± 0.80, and 14.41 ± 0.76 g/dL, respectively. A slight decrease in the mean Hb level was observed at one month following denture insertion, followed by a progressive increase over time, ultimately exceeding the baseline value by the sixth month.

**Table 2:** Comparison of pretreatment (baseline) and postdenture hemoglobin levels (g/dL) at different follow-up periods among study participants (n = 30) Values are expressed as mean ± SD. Comparison of hemoglobin levels across different time periods (baseline, one month, three months, and six months after complete denture insertion) was performed using one-way analysis of variance. A p value of <0.05 was considered statistically significant SD: standard deviation

Time period	Mean ± SD (n = 30)	F value	p value
Baseline	13.14 ± 0.81	18.48	<0.001
1 month	13.05 ± 0.82		
3 months	13.69 ± 0.80		
6 months	14.41 ± 0.76		

Comparison of Hb levels across the different time intervals using ANOVA revealed a statistically significant difference among the study periods (F = 18.48, p < 0.001) (Table 2). This finding indicates a significant temporal change in Hb levels following complete denture insertion.

Further pairwise comparisons of mean Hb levels across time periods were performed using the Tukey post hoc test. The analysis revealed no statistically significant difference (p > 0.05) in Hb levels between baseline and one month following denture insertion (Table 3). However, Hb levels were significantly higher at three and six months compared with baseline and one month (p < 0.05 to p < 0.001). In addition, a statistically significant increase (p < 0.01) in Hb levels was also observed at six months compared to three months, indicating a progressive improvement over time. At the final evaluation (six months), the net mean increase in Hb level, calculated as the change from baseline to six months, was 8.8%, suggesting a notable improvement in Hb status following complete denture rehabilitation.

**Table 3:** Pairwise comparison of mean hemoglobin levels (g/dL) between different time periods using Tukey post hoc test (n = 30) Pairwise comparisons of mean hemoglobin levels across different time periods (baseline, one month, three months, and six months after complete denture insertion) were performed using the Tukey post hoc test following ANOVA. Values represent the mean difference between groups with corresponding Tukey q statistics and 95% CIs. A p value of <0.05 was considered statistically significant The q value is obtained using the Tukey test statistic CI: confidence interval; ANOVA: analysis of variance

Comparison	Mean difference	q value	p value	95% CI of difference
Baseline vs. 1 month	0.09	0.64	>0.05	-0.445 to 0.631
Baseline vs. 3 months	0.55	3.77	<0.05	0.012-1.088
Baseline vs. 6 months	1.27	8.71	<0.001	0.732-1.808
1 vs. 3 months	0.64	4.41	<0.05	0.106-1.181
1 vs. 6 months	1.36	9.35	<0.001	0.826-1.901
3 vs. 6 months	0.72	4.94	<0.01	0.182-1.258

The pretreatment (baseline) and postdenture serum calcium levels at one, three, and six months are summarized (Table 4). The mean serum calcium levels at baseline, one month, three months, and six months were 8.54 ± 0.44, 8.29 ± 0.50, 8.88 ± 0.50, and 9.28 ± 0.48 mg/dL, respectively. A slight decline in the mean serum calcium level was observed at one month following denture insertion, followed by a progressive increase over time, ultimately exceeding the baseline value by the sixth month. Comparison of serum calcium levels across the different time periods using ANOVA demonstrated a statistically significant difference among the study periods (F = 24.13, p < 0.001) (Table 4), indicating a significant temporal change in serum calcium levels after complete denture rehabilitation.

**Table 4:** Comparison of pretreatment (baseline) and postdenture serum calcium levels (mg/dL) at different follow-up periods among study participants (n = 30) Values are expressed as mean ± SD. Comparison of serum calcium levels at baseline and at one, three, and six months after complete denture insertion was performed using one-way ANOVA. A p value of <0.05 was considered statistically significant SD: standard deviation; ANOVA: analysis of variance

Time period	Mean ± SD (n = 30)	F value	p value
Baseline	8.54 ± 0.44	24.13	<0.001
1 month	8.29 ± 0.50		
3 months	8.88 ± 0.50		
6 months	9.28 ± 0.48		

Further pairwise comparisons of mean serum calcium levels across time periods were performed using the Tukey post hoc test. The analysis demonstrated no statistically significant difference (p > 0.05) in serum calcium levels between baseline and one month following denture insertion (Table 5). However, serum calcium levels were significantly higher at three and six months compared with baseline and one month (p < 0.05 to p < 0.001). Furthermore, a statistically significant increase (p < 0.01) in serum calcium levels was also observed at six months compared with three months, indicating a progressive improvement over the follow-up period. At the final evaluation (six months), the net mean increase in serum calcium level, calculated as the change from baseline to six months, was 8.0%, suggesting a notable improvement in serum calcium status following complete denture rehabilitation.

**Table 5:** Pairwise comparison of mean serum calcium levels (mg/dL) between different time periods using Tukey post hoc test (n = 30) Pairwise comparisons of mean serum calcium levels between different time periods (baseline, one month, three months, and six months after complete denture insertion) were performed using the Tukey post hoc test following ANOVA. Values represent mean differences with corresponding Tukey q statistics and 95% CIs. A p value of <0.05 was considered statistically significant The q value is obtained using the Tukey test statistic CI: confidence interval; ANOVA: analysis of variance

Comparison	Mean difference	q value	p value	95% CI of difference
Baseline vs. 1 month	0.25	2.82	>0.05	0.076-0.571
Baseline vs. 3 months	0.34	3.87	<0.05	0.016-0.662
Baseline vs. 6 months	0.74	8.50	<0.001	0.422-1.068
1 vs. 3 months	0.59	6.69	<0.001	0.263-0.910
1 vs. 6 months	0.99	11.32	<0.001	0.669-1.315
3 vs. 6 months	0.41	4.63	<0.01	0.083-0.729

The pretreatment (baseline) and postdenture vitamin B12 levels at one, three, and six months are summarized in Table 6. The mean vitamin B12 levels at baseline, one month, three months, and six months were 342.93 ± 72.66, 337.43 ± 72.33, 393.87 ± 70.23, and 444.53 ± 69.98 pg/mL, respectively. A slight decline in the mean vitamin B12 level was observed at one month following denture insertion, followed by a gradual increase over time, ultimately exceeding the baseline value by the sixth month. Comparison of vitamin B12 levels across the different time periods using ANOVA demonstrated a statistically significant difference among the study periods (F = 14.83, p < 0.001) (Table 6), indicating a significant temporal change in vitamin B12 levels after complete denture rehabilitation.

**Table 6:** Comparison of pretreatment (baseline) and postdenture vitamin B12 levels (pg/mL) at different follow-up periods among study participants (n = 30) Values are expressed as mean ± SD. Comparison of vitamin B12 levels at baseline and at one, three, and six months after complete denture insertion was performed using one-way ANOVA. A p value of <0.05 was considered statistically significant SD: standard deviation; ANOVA: analysis of variance

Time period	Mean ± SD (n = 30)	F value	p value
Baseline	342.93 ± 72.66	14.83	<0.001
1 month	337.43 ± 72.33		
3 months	393.87 ± 70.23		
6 months	444.53 ± 69.98		

Further pairwise comparisons of mean vitamin B12 levels across time periods were performed using the Tukey post hoc test. The analysis demonstrated no statistically significant difference (p > 0.05) in vitamin B12 levels between baseline and one month following denture insertion (Table 7). However, vitamin B12 levels were significantly higher at three and six months compared with baseline and one month (p < 0.05 to p < 0.001). Furthermore, a statistically significant increase (p < 0.05) in vitamin B12 levels was also observed at six months compared with three months, indicating a progressive improvement over the follow-up period. At the final evaluation (six months), the net mean increase in vitamin B12 level, calculated as the change from baseline to six months, was 22.9%, suggesting a substantial improvement in vitamin B12 status following complete denture rehabilitation.

**Table 7:** Pairwise comparison of mean vitamin B12 levels (pg/mL) between different time periods using Tukey post hoc test (n = 30) Pairwise comparisons of mean vitamin B12 levels between different time periods (baseline, one month, three months, and six months after complete denture insertion) were performed using the Tukey post hoc test following ANOVA. Values represent mean differences with corresponding Tukey q statistics and 95% CIs. A p value of <0.05 was considered statistically significant The q value is obtained using the Tukey test statistic CI: confidence interval; ANOVA: analysis of variance

Comparison	Mean difference	q value	p value	95% CI of difference
Baseline vs. 1 month	5.50	0.42	>0.05	-42.52 to 53.52
Baseline vs. 3 months	50.93	3.91	<0.05	2.91-98.95
Baseline vs. 6 months	101.60	7.80	<0.001	53.58-149.60
1 vs. 3 months	56.43	4.34	<0.05	8.41-104.50
1 vs. 6 months	107.10	8.23	<0.001	59.08-155.10
3 vs. 6 months	50.67	3.89	<0.05	2.65-98.69

The pretreatment (baseline) and postdenture serum HDL levels at one, three, and six months are summarized in Table 8. The mean serum HDL levels at baseline, one month, three months, and six months were 39.65 ± 4.22, 39.22 ± 4.14, 42.40 ± 3.98, and 43.56 ± 3.93 mg/dL, respectively. A slight decrease in the mean serum HDL level was observed at one month following denture insertion, followed by a gradual increase over time, ultimately exceeding the baseline value by the sixth month. Comparison of serum HDL levels across the different time periods using ANOVA revealed a statistically significant difference among the study periods (F = 8.06, p < 0.001) (Table 8), indicating a significant temporal change in serum HDL levels after complete denture rehabilitation.

**Table 8:** Comparison of pretreatment (baseline) and postdenture serum HDL levels (mg/dL) at different follow-up periods among study participants (n = 30) Values are presented as mean ± SD. Comparison of serum HDL levels at baseline and at one, three, and six months after complete denture insertion was performed using one-way ANOVA. A p value of <0.05 was considered statistically significant SD: standard deviation; ANOVA: analysis of variance; HDL: high-density lipoprotein

Time period	Mean ± SD (n = 30)	F value	p value
Baseline	39.65 ± 4.22	8.06	<0.001
1 month	39.22 ± 4.14		
3 months	42.40 ± 3.98		
6 months	43.56 ± 3.93		

Further pairwise comparisons of mean serum HDL levels across time periods were performed using the Tukey post hoc test. The analysis revealed no statistically significant difference (p > 0.05) in serum HDL levels between baseline and one month following denture insertion (Table 9). However, serum HDL levels were significantly higher at three and six months compared with baseline and one month (p < 0.05 to p < 0.001). In contrast, no statistically significant difference (p > 0.05) was observed between three and six months, indicating that the serum HDL levels at these two follow-up periods were comparable. At the final evaluation (six months), the net mean increase in serum HDL level, calculated as the change from baseline to six months, was 9.0%, suggesting an overall improvement in serum HDL levels following complete denture rehabilitation.

**Table 9:** Pairwise comparison of mean serum HDL levels (mg/dL) between different time periods using Tukey post hoc test (n = 30) Pairwise comparisons of mean serum HDL levels across different time periods (baseline, one month, three months, and six months after complete denture insertion) were performed using the Tukey post hoc test following ANOVA. Values represent mean differences with corresponding Tukey q statistics and 95% CIs. A p value of <0.05 was considered statistically significant The q value is obtained using the Tukey test statistic CI: confidence interval; HDL: high-density lipoprotein; ANOVA: analysis of variance

Comparison	Mean difference	q value	p value	95% CI of difference
Baseline vs. 1 month	0.43	0.58	>0.05	-2.309 to 3.171
Baseline vs. 3 months	2.75	3.70	<0.05	0.008-5.487
Baseline vs. 6 months	3.91	5.27	<0.01	1.172-6.651
1 vs. 3 months	3.18	4.28	<0.05	0.439-5.918
1 vs. 6 months	4.34	5.85	<0.001	1.603-7.082
3 vs. 6 months	1.16	1.57	>0.05	1.576-3.904

The pretreatment (baseline) and postdenture BMI levels at one, three, and six months are summarized in Table 10. The mean BMI values at baseline, one month, three months, and six months were 18.17 ± 1.77, 18.06 ± 1.82, 19.65 ± 1.96, and 21.01 ± 1.98 kg/m², respectively. A slight decrease in mean BMI was observed at one month following denture insertion, followed by a progressive increase over time, eventually exceeding the baseline value by the sixth month. Comparison of BMI levels across the different time periods using ANOVA demonstrated a statistically significant difference among the study periods (F = 16.39, p < 0.001) (Table 10), indicating a significant temporal change in BMI following complete denture rehabilitation.

**Table 10:** Comparison of pretreatment (baseline) and postdenture BMI levels (kg/m²) at different follow-up periods among study participants (n = 30) Values are presented as mean ± SD. Comparison of BMI levels at baseline and at one, three, and six months after complete denture insertion was performed using one-way ANOVA. A p value of <0.05 was considered statistically significant SD: standard deviation; BMI: body mass index; ANOVA: analysis of variance

Time period	Mean ± SD (n = 30)	F value	p value
Baseline	18.17 ± 1.77	16.39	<0.001
1 month	18.06 ± 1.82		
3 months	19.65 ± 1.96		
6 month	21.01 ± 1.98		

Further pairwise comparisons of mean BMI levels across time periods were performed using the Tukey post hoc test. The analysis revealed no statistically significant difference (p > 0.05) in BMI levels between baseline and one month following denture insertion (Table 11). However, BMI levels were significantly higher at three and six months than at baseline and one month (p < 0.05 to p < 0.001). Furthermore, a statistically significant increase in BMI (p < 0.05) was observed at six months compared with three months, indicating a progressive improvement over the follow-up period. At the final evaluation (six months), the net mean gain in BMI, calculated as the change from baseline to six months, was 13.5%, suggesting a notable improvement in the nutritional status of the subjects following complete denture rehabilitation.

**Table 11:** Pairwise comparison of mean BMI levels (kg/m²) between different time periods using Tukey post hoc test (n = 30) Pairwise comparisons of mean BMI levels across different time periods (baseline, one month, three months, and six months after complete denture insertion) were performed using the Tukey post hoc test following ANOVA. Values represent mean differences with corresponding Tukey q statistics and 95% CIs. A p value of <0.05 was considered statistically significant The q value is obtained using the Tukey test statistic CI: confidence interval; BMI: body mass index; ANOVA: analysis of variance; CI: confidence interval

Time period	Mean difference	q value	p value	95% CI of difference
Baseline vs. 1 month	0.11	0.33	>0.05	-1.155 to 1.382
Baseline vs. 3 months	1.48	4.29	<0.05	0.207-2.744
Baseline vs. 6 months	2.83	8.24	<0.001	1.564-4.101
1 vs. 3 months	1.59	4.62	<0.01	0.320-2.857
1 vs. 6 months	2.95	8.57	<0.001	1.678-4.215
3 vs. 6 months	1.36	3.95	<0.05	0.089-2.626

The change in nutritional status parameters Hb, serum calcium, vitamin B12, serum HDL, and BMI) from baseline to six months after complete denture insertion is summarized in Table 12. The net mean increase (gain) observed at the six-month follow-up compared with baseline was 1.27 ± 0.28 g/dL for Hb, 0.74 ± 0.31 mg/dL for serum calcium, 101.60 ± 10.99 pg/mL for vitamin B12, 3.91 ± 0.94 mg/dL for serum HDL, and 2.83 ± 1.00 kg/m² for BMI. Among these parameters, the maximum improvement was observed in vitamin B12 levels, whereas the least increase was noted in serum calcium levels, indicating variable degrees of improvement in nutritional status following complete denture rehabilitation.

**Table 12:** Change in nutritional status parameters of subjects at six months after complete denture insertion (n = 30) Values represent the mean change (six months to baseline) ± SD in nutritional status parameters among study participants following complete denture insertion. Positive values indicate an increase in the respective parameter at six months compared with baseline SD: standard deviation; HDL: high-density lipoprotein; BMI: body mass index

Variable	Mean ± SD (n = 30)
Hemoglobin (g/dL)	1.27 ± 0.28
Serum calcium (mg/dL)	0.74 ± 0.31
Vitamin B12 (pg/mL)	101.60 ± 10.99
Serum HDL (mg/dL)	3.91 ± 0.94
BMI (kg/m^2^)	2.83 ± 1.00

The intercorrelations among changes in the nutritional status parameters Hb, serum calcium, vitamin B12, serum HDL, and BMI of the study participants are summarized in Table 13. Pearson’s correlation analysis demonstrated weak and nonsignificant correlations (p > 0.05) among most nutritional parameters. This indicates that the changes in these nutritional indicators occurred independently of each other. However, a statistically significant negative (inverse) correlation was observed between BMI and Hb levels (r = -0.38, p < 0.05). This finding suggests that an increase in BMI was associated with a decrease in Hb levels, and vice versa. Overall, the results indicate that although improvements in nutritional parameters were observed following complete denture rehabilitation, these changes were largely independent, except for the inverse relationship between BMI and Hb.

**Table 13:** Intercorrelation of changes in nutritional status parameters of subjects using Pearson correlation analysis (n = 30) The table presents the Pearson correlation coefficients (r) indicating the interrelationship among the changes in nutritional status parameters (hemoglobin, serum calcium, vitamin B12, serum HDL, and BMI) of the subjects from baseline to six months after complete denture insertion ns indicates not significant (p > 0.05) ^*^Statistically significant (p < 0.05) HDL: high-density lipoprotein; BMI: body mass index

Variable	Hemoglobin	Serum calcium	Vitamin B12	Serum HDL	BMI
Hemoglobin	1.00	-	-	-	-
Serum calcium	-0.13^ns^	1.00	-	-	-
Vitamin B12	-0.17^ns^	0.02^ns^	1.00	-	-
Serum HDL	0.18^ns^	-0.24^ns^	-0.24^ns^	1.00	-
BMI	-0.38^*^	-0.17^ns^	-0.11^ns^	-0.02^ns^	1.00

The changes in nutritional status parameters (Hb, serum calcium, vitamin B12, serum HDL, and BMI) of the subjects according to age groups (≤60 years and >60 years) are summarized in Table 14. The mean change in most nutritional parameters was higher among subjects aged ≤60 years than among those aged >60 years, except for Hb levels. However, the differences in the mean changes between the two age groups were not statistically significant (p > 0.05). These findings suggest that the observed improvement in nutritional status following complete denture insertion was comparable across both age groups, indicating that the gain in nutritional parameters may not be significantly influenced by age.

**Table 14:** Change in nutritional status parameters of subjects according to age groups (n = 30) The table presents the change in nutritional status parameters of subjects from baseline to six months after complete denture insertion, stratified according to age groups (≤60 years and >60 years). Values are expressed as mean ± SD. Comparison between the two age groups was performed using the Student’s t-test, and the results are presented as t values with corresponding p values HDL: high-density lipoprotein; BMI: body mass index; SD: standard deviation

Variable	Age ≤ 60 years (n = 12)	Age > 60 years (n = 18)	Mean difference	t value	p value
Hemoglobin (g/dL)	1.20 ± 0.22	1.32 ± 0.31	0.12	1.11	0.276
Serum calcium (mg/dL)	0.75 ± 0.28	0.74 ± 0.33	0.01	0.06	0.949
Vitamin B12 (pg/mL)	101.75 ± 10.62	101.50 ± 11.54	0.25	0.06	0.953
Serum HDL (mg/dL)	4.08 ± 1.01	3.80 ± 0.89	0.28	0.79	0.434
BMI (kg/m^2^)	3.06 ± 0.77	2.68 ± 1.13	0.38	1.04	0.309

The change in nutritional status parameters (Hb, serum calcium, vitamin B12, serum HDL, and BMI) of the subjects according to the duration of edentulism (≤15 and >15 months) is summarized in Table 15. The mean change in most nutritional parameters was higher among subjects with a shorter duration of edentulism (≤15 months) than among those with a longer duration (>15 months), except for Hb levels. However, the differences in the mean changes between the two groups were not statistically significant (p > 0.05). These findings suggest that the improvement in nutritional status following complete denture insertion was comparable between the two groups, indicating that the gain in nutritional parameters may not be significantly influenced by the duration of edentulism.

**Table 15:** Change in nutritional status parameters of subjects according to duration of edentulism (n = 30) The table presents the change in nutritional status parameters of the subjects from baseline to six months after complete denture insertion, stratified according to the duration of edentulism (≤15 and >15 months). Values are expressed as mean ± SD. Comparison between the two groups was performed using the Student’s t-test, and the results are presented as t values with corresponding p values HDL: high-density lipoprotein; BMI: body mass index; SD: standard deviation

Variable	Duration of edentulous ≤15 months (n = 15)	Duration of edentulous >15 months (n = 15)	Mean difference	t value	p value
Hemoglobin (g/dL)	1.19 ± 0.24	1.35 ± 0.30	0.16	1.66	0.108
Serum calcium (mg/dL)	0.75 ± 0.34	0.74 ± 0.28	0.01	0.11	0.917
Vitamin B12 (pg/mL)	102.00 ± 12.32	101.20 ± 9.91	0.80	0.20	0.846
Serum HDL (mg/dL)	4.06 ± 1.00	3.76 ± 0.87	0.30	0.88	0.387
BMI (kg/m^2^)	3.04 ± 1.05	2.63 ± 0.94	0.41	1.13	0.266

## Discussion

Teeth play a crucial role in the process of food intake and digestion. They assist in cutting, tearing, and grinding food and facilitate its retention within the oral cavity during mastication. During this phase, food is not only mechanically processed but also mixed with saliva containing digestive enzymes, thereby initiating digestion. Loss of natural dentition compromises this process and may consequently influence dietary intake and nutritional status.

Aging is accompanied by several physiological changes that may adversely affect oral and systemic health. After the age of 60 years, skeletal changes, including bone resorption, become more evident, particularly in women following menopause due to altered calcium metabolism. These changes contribute to tooth loss and alveolar bone resorption. As a result, edentulism becomes one of the most prevalent oral health problems in the elderly population and may significantly influence dietary habits and nutritional well-being.

Previous studies have demonstrated that tooth loss and denture wearing may influence food selection. Allen and McMillan, and Tajbakhsh et al. reported that denture wearers were less likely to consume vegetables, whole-meal bread, and dietary fiber compared with individuals retaining natural teeth [14,15]. Similarly, Allen and McMillan, and Isaksson et al. observed that edentulous individuals tend to consume softer foods than dentate individuals, thereby limiting the variety of nutrient-rich foods in their diet [14,16]. Consequently, individuals with natural dentition generally exhibit a more diverse diet and better nutritional status.

Nutritional status in the elderly is influenced by a complex interaction of physiological, psychosocial, and oral factors. Physiologically, aging is associated with a gradual reduction in lean body mass, particularly skeletal muscle mass (sarcopenia). Since muscle mass influences strength, mobility, metabolic rate, and insulin sensitivity, its decline may adversely affect nutritional status. Psychosocial factors also contribute significantly; elderly individuals living alone, experiencing social isolation, or facing financial constraints due to retirement and rising healthcare costs are more vulnerable to nutritional deficiencies.

Oral health itself plays a critical role in determining nutritional status. According to Palmer, nutrition and oral health share a bidirectional relationship [17]. Oral conditions such as tooth loss or poorly fitting dentures may reduce chewing ability and limit the intake of certain foods, particularly hard or fibrous foods. Consequently, edentulous individuals often develop fixed dietary habits and may exclude several nutritious food items from their diet.

Edentulous patients frequently avoid foods requiring extensive mastication, such as nuts, leafy vegetables, and certain dairy products. Foods rich in vitamin B12, including meat, poultry, and fish, also require efficient mastication for comfortable consumption. Deficiency of vitamin B12 can lead to megaloblastic anemia and neurological complications such as numbness, cognitive impairment, and memory disturbances. Similarly, reduced iron intake may contribute to anemia characterized by fatigue, impaired immunity, and delayed wound healing, which may affect denture adaptation. Other nutritional parameters, including calcium, HDL cholesterol, and BMI, are also important indicators of overall nutritional status and systemic health.

Considering these relationships, the present study aimed to evaluate the effect of complete denture rehabilitation on selected nutritional biomarkers, including Hb, serum calcium, vitamin B12, serum HDL, and BMI in completely edentulous patients.

Thirty completely edentulous patients who were first-time denture wearers and had been edentulous for at least one year were included in the study. All participants were systemically healthy and belonged to the same SES to minimize confounding variables. Biochemical investigations were performed at four time points: prior to denture insertion (baseline) and at one, three, and six months following denture insertion.

Anthropometric assessment of nutritional status traditionally includes measurements such as BMI and body weight [12]. In the present study, the results demonstrated a consistent improvement in all nutritional parameters following complete denture rehabilitation, with most statistically significant changes becoming evident from the third month onward.

Vignesh et al. similarly reported that edentulous patients exhibited lower levels of Hb, serum calcium, serum HDL, and BMI prior to denture insertion, suggesting that tooth loss may adversely affect systemic nutritional parameters [4]. Following prosthetic rehabilitation, these parameters showed significant improvement.

The present study observed that Hb levels initially showed a slight decline at one month following denture insertion, but increased significantly by three and six months. Overall, Hb levels increased by 1.27 g/dL (8.8%) at six months. The early decline may reflect the adaptation period during which patients adjust to their new dentures and may temporarily experience reduced chewing efficiency. During the initial phase, patients are often advised to limit denture use during mastication and gradually adapt to bilateral chewing. As adaptation improves, patients regain the ability to consume iron-rich foods, such as leafy vegetables, legumes, and meats. Similar findings were reported by Agrawal et al., who observed minimal changes during the first month but significant nutritional improvement after three months of denture use [2].

Serum calcium levels also demonstrated a significant increase from the third month onward, with a net gain of 0.74 mg/dL (8%) at six months. Calcium intake is largely dependent on foods such as dairy products and leafy vegetables, which often require adequate masticatory efficiency. Restoration of chewing ability through dentures likely enabled patients to reintroduce these foods into their diet [18].

Vitamin B12 levels showed the greatest improvement among all biochemical parameters, increasing by 22.9% at six months. This substantial increase may be attributed to improved consumption of animal-based foods such as meat, eggs, and fish, which are primary dietary sources of vitamin B12. Since vitamin B12 deficiency is relatively common among elderly individuals and is associated with neurological and hematological complications, the observed improvement highlights the broader health benefits of oral rehabilitation [18].

Serum HDL levels also increased significantly following denture insertion, showing a net improvement of 9.0%. HDL is considered a protective lipoprotein associated with cardiovascular health [19]. The improvement may be related to a more balanced diet including fiber-rich foods, nuts, and healthy fats, which edentulous patients often avoid before prosthetic rehabilitation.

BMI values also showed a significant increase over time, with a net gain of 2.83 kg/m² (13.5%). Improved masticatory efficiency likely enhanced caloric intake and dietary satisfaction, contributing to weight gain and better nutritional status. Madhuri et al. reported similar findings, demonstrating significant improvement in BMI following complete denture rehabilitation [11].

The study further evaluated the influence of age and duration of edentulism on nutritional improvement. Neither age nor duration of edentulism demonstrated a statistically significant association with the magnitude of nutritional improvement. These findings suggest that complete denture therapy can benefit patients irrespective of age group or the duration of tooth loss.

Interestingly, although patients with a shorter duration of edentulism generally showed slightly greater improvements in nutritional parameters, these differences were not statistically significant. This suggests that even individuals with long-standing edentulism may experience meaningful nutritional recovery once masticatory function is restored.

Overall, the findings of this study highlight the important relationship between oral rehabilitation and systemic health. Restoration of masticatory efficiency through complete dentures may improve dietary intake, nutritional biomarkers, and overall quality of life in elderly patients.

Clinically, these findings emphasize that complete denture therapy should be viewed not only as a prosthetic intervention but also as an important component of systemic health management. Nutritional counseling may further enhance the benefits of denture therapy. In addition, monitoring nutritional biomarkers in elderly dental patients could contribute to more comprehensive patient care.

Limitations of the study

Despite the encouraging findings, certain limitations of the present study should be acknowledged. The study was restricted to male participants to minimize gender-related physiological and hormonal variability; however, this limits the generalizability of the findings to female populations. The relatively small sample size (n = 30) may further limit the external validity of the results. Although participants with both underweight and normal BMI were included to reflect real-world clinical variability, this may introduce potential bias in interpreting nutritional outcomes.

Additionally, dietary intake was not directly assessed using standardized methods such as dietary recall or food diaries, and the type of diet (e.g., vegetarian or nonvegetarian) was not recorded, both of which could have influenced the nutritional parameters evaluated. The absence of standardized nutritional assessment tools (e.g., validated screening indices) before enrolment is another limitation. Furthermore, individual variability in adaptation to complete dentures may have influenced the observed nutritional outcomes. Finally, only a limited number of biochemical markers were evaluated; inclusion of a broader panel of nutritional indicators could provide a more comprehensive assessment of nutritional status.

## Conclusions

Complete denture rehabilitation significantly improves the nutritional status of completely edentulous geriatric patients by restoring masticatory function and facilitating a more balanced diet. The present study demonstrated improvements in both anthropometric and biochemical parameters, with BMI showing a consistent increase after insertion, supporting its utility as a simple and reliable indicator of nutritional status when interpreted alongside markers such as Hb, serum calcium, HDL, and vitamin B12. These findings indicate that prosthetic rehabilitation contributes positively to systemic health and quality of life, irrespective of age and duration of edentulism, and should be considered an integral component of comprehensive geriatric care. However, further studies with larger and more diverse populations, the inclusion of both genders, the use of standardized nutritional assessment tools, detailed dietary evaluation, and a broader range of biochemical markers are recommended to enhance the validity and applicability of these findings.
